# Decreased Cerebral Amyloid-β Depositions in Patients With a Lifetime History of Major Depression With Suspected Non-Alzheimer Pathophysiology

**DOI:** 10.3389/fnagi.2022.857940

**Published:** 2022-05-26

**Authors:** Kuan-Yi Wu, Kun-Ju Lin, Chia-Hsiang Chen, Chia-Yih Liu, Yi-Ming Wu, Cheng-Sheng Chen, Tzu-Chen Yen, Ing-Tsung Hsiao

**Affiliations:** ^1^Department of Psychiatry, Chang Gung Memorial Hospital, College of Medicine, Chang Gung University, Taoyuan City, Taiwan; ^2^Department of Nuclear Medicine, Center for Advanced Molecular Imaging and Translation, Chang Gung Memorial Hospital, Taoyuan City, Taiwan; ^3^Department of Medical Imaging and Radiological Sciences, College of Medicine and Healthy Aging Research Center, Chang Gung University, Taoyuan City, Taiwan; ^4^Neuroscience Research Center, Chang Gung Memorial Hospital, Linkou Medical Center, Taoyuan City, Taiwan; ^5^Department of Radiology, Chang Gung Memorial Hospital, Taoyuan City, Taiwan; ^6^Department of Psychiatry, Kaohsiung Medical University Hospital, College of Medicine, Kaohsiung Medical University, Kaohsiung City, Taiwan

**Keywords:** suspected non-Alzheimer pathophysiology (SNAP), major depressive disorder (MDD), amyloid-β (Aβ), ^18^F-florbetapir (AV-45/Amyvid), neurodegeneration, depression in old age

## Abstract

Cerebral amyloid-β (Aβ) depositions in depression in old age are controversial. A substantial proportion of individuals with late-life major depressive disorder (MDD) could be classified as having suspected non-Alzheimer’s disease pathophysiology (SNAP) by a negative test for the biomarker amyloid-β (Aβ−) but positive neurodegeneration (ND+). This study aimed to evaluate subthreshold Aβ loads in amyloid-negative MDD, particularly in SNAP MDD patients. This study included 46 amyloid-negative MDD patients: 23 SNAP (Aβ−/ND+) MDD and 23 Aβ−/ND− MDD, and 22 Aβ−/ND− control subjects. All subjects underwent ^18^F-florbetapir PET, FDG-PET, and MRI. Regions of interest (ROIs) and voxel-wise group comparisons were performed with adjustment for age, gender, and level of education. The SNAP MDD patients exhibited significantly deceased ^18^F-florbetapir uptakes in most cortical regions but not the parietal and precuneus cortex, as compared with the Aβ−/ND− MDD and control subjects (FDR correction, *p* < 0.05). No correlations of neuropsychological tests or depression characteristics with global cortical uptakes, but significant positive correlations between cognitive functions and adjusted hippocampal volumes among different groups were observed. The reduced Aβ depositions in the amyloid-negative MDD patients might be attributed mainly to the SNAP MDD patients. Our results indicated that meaningfully low amounts of subclinical Aβ might contain critical information on the non-amyloid-mediated pathogenesis.

## Introduction

Converging evidence from multiple meta-analyses (Jorm, 2001; Ownby et al., 2006; Diniz et al., 2013) suggests that depression approximately doubles an individual’s risk of developing dementia later in life. Brain amyloid-β (Aβ) deposition serves as a gold-standard hallmark of pathogenesis in Alzheimer’s disease (AD). Early autopsy studies (Rapp et al., 2006) have shown more pronounced Aβ plaque in AD patients who have lifetime major depressive disorder (MDD) as compared with those without MDD. Depression in old age has been increasingly investigated in terms of the relationship with *in vivo* cerebral Aβ via validated amyloid imaging in the past decade. However, the results regarding cerebral Aβ amounts in depression have been inconsistent and controversial. Butters et al. (2008) and Wu et al. (2014) found that MDD patients have increased cortical Aβ in comparison with non-depressed healthy controls; however, Madsen et al. (2012) found no difference between midlife MDD and cognitively normal individuals. Unexpectedly, a recent study by Mackin et al. (2021) showed an even more reduced cortical Aβ level in depressed elderly patients as compared with non-depressed cognitively normal subjects. Therefore, to date, related studies have yielded variable and conflicting results.

Depression in old age, not surprisingly, might represent an etiological entity with both clinical and pathophysiological heterogeneity. Our previous study (Wu et al., 2018) provided evidence of the diversity of involved neurodegenerative processes in elderly depressed individuals. We found that some depressed elderly patients entered the AD prodrome; others might be subject to a neurodegenerative pathway completely distinct from AD. In the past, we have always paid more attention to amyloid positivity for an accurate diagnosis of AD; however, the impact of subthreshold Aβ is gradually being explored (Bischof and Jacobs, 2019). Recent studies reported subthreshold Aβ and Aβ accumulation rate could predict early tau deposition in those who were nominally amyloid negative (Leal et al., 2018). Additionally, non-amyloid-mediated neurodegeneration could be associated with subthreshold Aβ changes (Jack et al., 2013). Subthreshold Aβ might provide clinically meaningful and useful information that may reflect various individual neurodegenerative processes. To date, a subthreshold Aβ condition among amyloid-negative MDD patients remains largely unclear. In order to make group comparisons on the same basis of amyloid negativity status, both samples of amyloid-negative MDD and control subjects were included in this study.

Among the amyloid-negative individuals, a suspected non-Alzheimer disease pathophysiology (SNAP) can be indicated by a negative test for β-amyloid (Aβ−) but a positive test for neurodegeneration (ND+). Hippocampal atrophy and glucose hypometabolism within AD-vulnerable regions have been widely adopted as ND biomarkers (Jack et al., 2012; Caroli et al., 2015; Mormino et al., 2016). In this study, amyloid-negative MDD patients were further classified into SNAP (Aβ−/ND+) MDD and Aβ−/ND− MDD groups. This study aimed to investigate the subthreshold Aβ characteristics in amyloid-negative MDD subjects, particularly in SNAP MDD patients, utilizing ^18^F-florbetapir PET imaging.

## Materials and Methods

### Subjects

This prospective, cross-sectional study, performed at Chang Gung Memorial Hospital Geriatric Psychiatry Center from July 2015 to June 2017, enrolled 50 MDD patients and 12 non-depressed control subjects. Of the enrolled subjects, 4 MDD patients and 1 control subject were excluded due to amyloid-positive results on ^18^F-florbetapir PET scanning. To increase the control sample size, another 11 control subjects were recruited from the Taiwan-Alzheimer’s Disease Neuroimaging Initiative (T-ADNI) study cohort (Lin et al., 2016) owing to the availability of complete information with regards to the Aβ−/ND− profile in that cohort. Thus, a total of 46 amyloid-negative MDD and 22 Aβ−/ND− control subjects were included in the study.

Each MDD patient was assessed for the presence of lifetime major depressive episodes according to the DSM-IV [DSM-5 (American Psychiatric Association, 2013) after 2016] via a clinical structured interview and retrospective medical chart review. The lifetime course of major depression was also clarified, including age at onset of major depression, number of major depressive episodes, late-onset major depression (cut-off set at 60 years) and time since onset of first depression. Control subjects were confirmed as having an absence of lifetime psychiatric illnesses, and were deemed cognitively normal (MMSE ≥ 27 and CDR = 0). All subjects were aged > 50 years; had no definite neurologic diseases affecting brain structure (e.g., completed stroke, traumatic head injury or epilepsy); suffered no unstable medical diseases involving the heart, lungs, liver or kidneys; and did not have alcohol or other substance abuse currently or in the past 1 year. None of the subjects met the NIA-AA criteria for dementia due to AD (Jack et al., 2018), the IWG criteria for typical/atypical AD dementia (Dubois et al., 2014), or the DSM-5 criteria for any type of dementia (American Psychiatric Association, 2013).

All eligible subjects underwent scans of ^18^F-florbetapir PET, FDG-PET, and brain MRI. Apolipoprotein E (APOE) genotypes were determined by polymerase chain reaction (PCR) study, and vascular risk factors as defined by the Framingham Stroke Risk Score (FSRS) were also identified. Except for the 11 control subjects recruited from the T-ADNI study cohort, comprehensive neuropsychological tests were performed by all subjects as per our previous study, and the results are presented as standard z-scores transformed using regression-based norms, adjusted for age and level of education (Wu et al., 2016). Written informed consent was obtained from all subjects, and the study protocol was approved by the Institutional Review Boards of the Ministry of Health and Welfare and Chang Gung Medical Center.

Amyloid-negative results were evaluated according to visual rating criteria from ^18^F-florbetapir PET scans (Sabri et al., 2015), which were confirmed by the same experienced nuclear physician who was blind to the clinical data and imaging analysis of each subject. The adjusted hippocampal volume (HVa) atrophy, a cut-off value described previously (Wu et al., 2018), and glucose hypometabolism within AD-vulnerable regions, as defined by the FDG t-sum score (Herholz et al., 2002), were adopted as ND biomarkers. Subjects were classified as neurodegeneration-positive (ND+) when positive for one of HVa atrophy or glucose hypometabolism (cut-off value of 6,879 mm^3^ for HVa and 11,089.681 for FDG t-sum score). All control subjects met the Aβ−/ND− profile.

### Magnetic Resonance Imaging Acquisition and Data Preprocessing

T1-weighted MRI was performed for all subjects using a 3T Siemens Magnetom TIM Trio scanner on PET/MR (Siemens Medical Solutions, Malvern, PA, United States). An acquisition protocol using a sagittal T1-weighted magnetization prepared rapid acquisition gradient echo (MPRAGE) sequence was applied with the following acquisition parameters: Repetition Time (TR)/Echo Time (TE) = 2,600/3.12 ms, TI = 900 ms, flip angle = 13°, voxel size = 0.5 mm × 0.5 mm × 1.1 mm. Structural scans were processed using FreeSurfer version 5.3 image analysis software^1^ for total bilateral hippocampal and intracranial volumes (Dale et al., 1999). A linear-regression normalization method was applied (Voevodskaya et al., 2014), with the total bilateral hippocampal volume adjusted by the estimated total intracranial volume to obtain the adjusted hippocampal volume (HVa), as described in our previous study (Wu et al., 2018), to reduce inter-subject variability.

### Positron Emission Tomography Imaging Acquisition and Data Preprocessing

Radiosynthesis of ^18^F-florbetapir (Yao et al., 2010) and amyloid PET data acquisition followed the same procedures as previously described (Lin et al., 2010). During the study, each ^18^F-florbetapir PET scan (380 ± 5 MBq) at 50–60 min post-injection was obtained using a Biograph mMR PET/MR System (Siemens Medical Solutions). The 3-D OSEM PET reconstruction algorithm (three iterations, 21 subsets; Gaussian filter: 2 mm; zoom: 3) with MR-based attenuation correction, scatter and random corrections was applied to obtain PET images with a matrix size of 344 × 344 × 127 and a voxel size of 0.83 mm^3^ × 0.83 mm^3^ × 2.03 mm^3^.

^18^F-FDG data were acquired at 30–50 min post-injection with a dose of 374 ± 13 MBq using a Biograph mCT PET/CT system (Siemens Medical Solutions). PET images were reconstructed using the 3D ordered subsets expectation maximization reconstruction algorithm with the parameters of four iterations, 24 subsets, Gaussian filter 2 mm, Zoom 3. In addition, CT-based attenuation correction, scatter and random corrections were performed using the correction methods provided by the manufacturer. The resulting reconstructed images were of a matrix size of 400 × 400 × 109 and a voxel size of 0.68 mm^3^ × 0.68 mm^3^ × 2.03 mm^3^.

Subsequent image quantitation analysis was performed using PMOD image analysis software (version 3.7, PMOD Technologies Ltd., Zurich, Switzerland). PET images were spatially normalized to the Montreal Neurological Institute (MNI) MRI template (Hsiao et al., 2013) using an MR-based method. Standardized uptake value ratio (SUVR) images for ^18^F-florbetapir were generated using the whole cerebellum as the reference region for subsequent analysis.

### Statistical Analysis

Demographic and clinical data are expressed as means ± SD or absolute numbers with proportions for descriptive statistics. Continuous variables were analyzed by non-parametric statistics using the Kruskal-Wallis test with Dunn’s *post hoc* multiple comparison. Categorical data were analyzed using the *X*^2^ test (Fisher’s exact test for APOE4, given the small numbers). Regions of interest (ROIs) comparisons of ^18^F-florbetapir SUVRs were conducted using the Kruskal-Wallis test with Dunn’s *post hoc* analysis. In addition, analyses of covariance (ANCOVA) were conducted to compare regional ^18^F-florbetapir SUVRs among the three groups, with adjustment for age and years of education; pairwise differences among the adjusted means were further evaluated with Bonferroni correction. Partial correlations between global ^18^F-florbetapir SUVRs and the neuropsychological test data or depression characteristics were evaluated using Pearson correlation analysis, with adjustment for age, years of education and HVa. Statistical analyses were performed using IBM SPSS version 25.0 (IBM Corp., Armonk, NY, United States), and *p* < 0.05 was considered to indicate statistical significance.

Voxel-wise group comparisons were performed in Statistical Parametric Mapping 12 (SPM 12^2^) using an ANCOVA model with age, gender and level of education as covariates. Pairwise group contrasts were performed between the SNAP MDD, Aβ−/ND− MDD and control groups. To reduce the likelihood of volume effects on the results, both ROIs and voxel-wise analyses in the study were conducted with atrophy-corrected data using partial volume correction (PVC) (Gonzalez-Escamilla et al., 2017) unless otherwise specified. Furthermore, comparison results were corrected for multiple comparisons using a false discovery rate (FDR) correction at *p* values (p_*FDR*_) < 0.05 at the voxel level.

## Results

### Subjects

The demographic and clinical characteristics of each group are shown in Table 1. The groups did not differ significantly in age, gender, ApoEε4 carriers, and vascular risk factors. The SNAP MDD and Aβ−/ND− MDD patients had similar HAM-D scores, in addition to similar clinical depressive features (age at onset, disease duration, depressive episodes, and late-onset depression).

**TABLE 1:** Demographic and clinical characteristics of the SNAP MDD patients, Aβ−/ND− MDD patients, and control subjects.

Characteristic	SNAP (Aβ−/ND+) MDD (*n* = 23)	Aβ−/ND− MDD (*n* = 23)	Controls (*n* = 22)	*p* Value
**Age (years)^c^**				
Mean ± SD	65.3 ± 6.6	62.8 ± 4.0	64.1 ± 5.0	0.172
Female gender, *n* (%)	19 (82.6)	19 (82.6)	14 (63.6)	0.226
**Education (years)**				
Mean ± SD	8.3 ± 4.2*^a^	8.1 ± 3.6*^a^	11.9 ± 4.5	0.006
**HAM-D^e^**				
Mean ± SD	11.1 ± 6.0***^a^	9.3 ± 5.3**^a^	2.6 ± 1.9	<0.001
**MMSE**				
Mean ± SD	24.4 ± 4.2***^a^	26.47 ± 2.5*^a^	28.5 ± 1.1	<0.001
CDR 0.5, *n* (%)	17 (73.9)***^a^	10 (43.5)***^a^	0	<0.001
APOE4, *n* (%)^d^	5 (21.7)	2 (8.7)	1 (9.1)	0.538
**FSRS^d^**				
Mean ± SD	9.1 ± 4.3	9.6 ± 3.2	8.1 ± 1.5	0.317
**Age at onset (years)**				
Mean ± SD	53.9 ± 10.7	52.6 ± 9.7	–	0.904
**Duration since onset of depression (years)**				
Mean ± SD	11.3 ± 9.5	10.0 ± 9.6	–	0.552
**Number of depressive episodes**				
Mean ± SD	2.3 ± 1.3	2.0 ± 1.3	–	0.366
Late-onset MDD, *n* (%)	7 (30.4)	4 (17.4)	–	0.494
**Cognitive domain z-scores, Mean ± SD^d^**				
Executive function	−0.8 ± 0.7***^a,*b^	−0.2 ± 0.7	0.3 ± 0.4	<0.001
Memory	−1.0 ± 0.9**^a^	−0.5 ± 1.0	0.1 ± 0.6	0.004
Processing speed	−1.3 ± 0.9***^a^	−0.7 ± 0.9*^a^	0.3 ± 0.9	<0.001
Visuospatial function	−0.4 ± 1.1*^a^	0.2 ± 0.8	0.6 ± 0.6	0.016
Language	0.8 ± 0.8	1.1 ± 0.8	1.5 ± 0.9	0.058
Attention	−0.4 ± 0.8**^a^	0.1 ± 1.1	0.7 ± 1.0	0.012

*SNAP, suspected non-Alzheimer’s disease pathophysiology; MDD, major depressive disorder; ND, neurodegeneration; HAM-D, 17-item Hamilton Depression Rating Scale; MMSE, Mini Mental Status Examination; CDR, Clinical Dementia Rating; APOE 4, Apolipoprotein E ε4 carrier; FSRS, Framingham Stroke Risk Score. p Values denote the significance of differences among the SNAP MDD, Aβ−/ND− MDD and control groups using the Kruskal-Wallis test (continuous variables) or the x^2^ test (categorical variables).*

^a^Dunn’s post hoc analysis, significant difference between the SNAP MDD or Aβ−/ND− MDD group and the control subjects; *p < 0.05, **p < 0.01, ***p < 0.001.

^b^Dunn’s post hoc analysis, significant difference between the SNAP MDD patients and the Aβ−/ND− MDD patients; *p < 0.05.

*^c^Age at time of*

*^18^F-florbetapir PET scan.*

*^d^Data available for 11 control subjects only owing to recruitment of control subjects from different projects.*

In terms of neuropsychological testing, the SNAP MDD patients had greater cognitive deficits than the control subjects in all neuropsychological tests after *post hoc* analyses; the most severe deficits occurred in the executive function (*p* < 0.001) and processing speed domains (*p* < 0.001).

Of the 23 SNAP MDD patients, there were five subjects with only hippocampal atrophy, and 11 subjects with only hypometabolism, and seven subjects with the presence of both. The neurodegeneration biomarker distributions are displayed in the Supplementary Figure 1.

### Regions of Interest Group Comparisons

**FIGURE 1:**
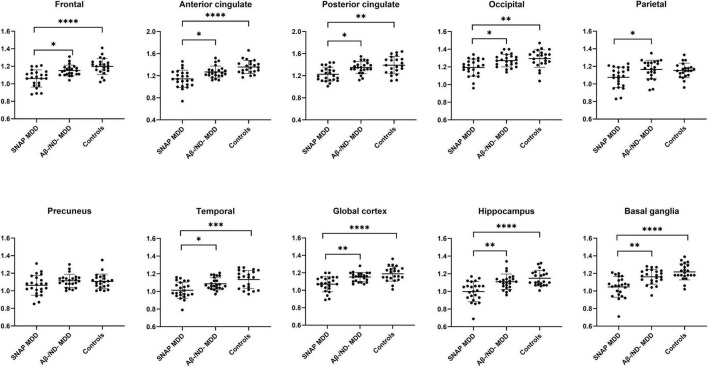
Regions of interest comparisons of ^18^F-florbetapir SUVRs in the SNAP (*n* = 23) and Aβ–/ND– MDD (*n* = 23) patients and controls (*n* = 22) using data of partial volume correction (PVC). *p < 0.05, ^**^p < 0.01, ^***^p < 0.001, ^****^p < 0.0001 using the Kruskal-Wallis test with Dunn’s *post hoc* analysis.

The 46 amyloid-negative MDD patients showed significantly decreased ^18^F-florbetapir SUVRs as compared with the controls in most cortices except the parietal and precuneus cortex (Supplementary Table 1). The three-group comparisons among the SNAP MDD, Aβ−/ND− MDD and control subjects are shown in Table 2. There were significant differences among the three groups in all ROIs assessed except the precuneus cortex. *Post hoc* analyses showed that, as compared with the controls, the SNAP MDD patients exhibited significantly decreased ^18^F-florbetapir uptakes in the frontal, anterior and posterior cingulate, occipital, temporal, hippocampus, basal ganglia and global cortex. Compared with the Aβ−/ND− MDD patients, the SNAP MDD patients showed ^18^F-florbetapir regions with greater decreases in the parietal cortex in addition to the ROIs observed in a *post hoc* comparison of the SNAP MDD and control subjects. No differences in ^18^F-florbetapir uptake were observed between the Aβ−/ND− MDD and control subjects in the *post hoc* ROI analyses. The differences among the three groups are shown in Figure 1. In order to confirm our findings, analyses were repeated using non-PVC original data; furthermore, ANCOVA analyses were conducted using PVC data with age and level of education as covariates. Comparable results were found, and are shown in Supplementary Tables 2, 3.

**TABLE 2:** Region of interest (ROI) group comparisons among the SNAP MDD, Aβ−/ND− MDD, and control groups.

Characteristic	SNAP (Aβ−/ND+) MDD	Aβ−/ND− MDD	Controls	*p* Value
	*n* = 23	*n* = 23	*n* = 22	
Frontal	1.06±0.10****^a,*b^	1.15 ± 0.06	1.20 ± 0.09	<0.0001
Anterior cingulate	1.14±0.16****^a,*b^	1.27 ± 0.11	1.36 ± 0.12	<0.0001
Posterior cingulate	1.23±0.13**^a,*b^	1.35 ± 0.11	1.39 ± 0.16	0.0008
Occipital	1.19±0.10**^a,*b^	1.27 ± 0.07	1.29 ± 0.10	0.0015
Parietal	1.08 ± 0.12*^b^	1.16 ± 0.11	1.15 ± 0.08	0.024
Precuneus	1.06 ± 0.11	1.11 ± 0.07	1.11 ± 0.08	0.1607
Temporal	1.01±0.09***^a,*b^	1.09 ± 0.07	1.14 ± 0.10	0.0004
Global cortex	1.07±0.09****^a,**b^	1.15 ± 0.05	1.19 ± 0.08	<0.0001
Hippocampus	1.00±0.11****^a,**b^	1.11 ± 0.09	1.15 ± 0.09	<0.0001
Basal ganglia	1.05±0.12****^a,**b^	1.16 ± 0.08	1.22 ± 0.09	<0.0001

*ROI, region of interest; SNAP, suspected non-Alzheimer’s disease pathophysiology; MDD, major depressive disorder; ND, neurodegeneration. p Values denote the significance of differences among the SNAP MDD, Aβ−/ND− MDD and control groups using the Kruskal-Wallis test.*

^a^Dunn’s post hoc analysis, significant difference between the SNAP MDD or Aβ−/ND− MDD group and the control subjects; *p < 0.05, **p < 0.01, ***p < 0.001, ****p < 0.0001.

^b^Dunn’s post hoc analysis, significant difference between the SNAP MDD patients and the Aβ−/ND− MDD patients; *p < 0.05, **p < 0.01.

### Voxel-Wise Group Comparisons

**FIGURE 2:**
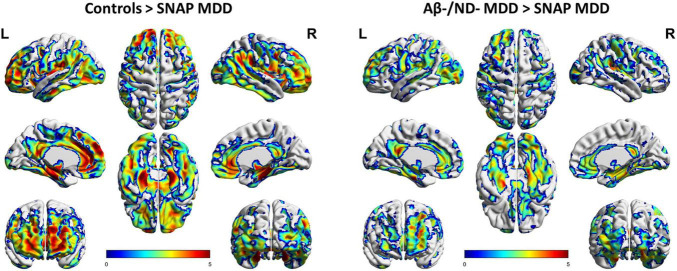
Voxel-wise comparisons of ^18^F-florbetapir uptakes in the SNAP (*n* = 23) and Aβ–/ND– MDD (*n* = 23) patients and controls (*n* = 22) using data of partial volume correction (PVC). Voxel-wise analyses were performed, including age, gender and level of education as covariates. Shown are T-maps using false discovery rate (FDR) correction for multiple comparisons (p_*FDR*_ < 0.05), with 3D brain renderings of ^18^F-florbetapir PET analysis on the ch2 template brain. Renders were created using BrainNet Viewer (https://www.nitrc.org/projects/bnv/).

The SNAP MDD patients exhibited much lower ^18^F-florbetapir uptakes than the control subjects in the lateral and medial frontal, anterior and posterior cingulate, temporoparietal junction, and occipital cortices, but this was not the case in the superior parietal and precuneus areas. The most prominent decreases in ^18^F-florbetapir SUVR were observed in the bilateral mesial temporal cortex and hippocampus (Figure 2, p_*FDR*_ < 0.05, adjusted for age, gender and level of education). Moreover, the SNAP MDD patients showed significantly decreased ^18^F-florbetapir retention as compared with the Aβ−/ND− MDD patients in similar areas to varying degrees, involving the frontal, anterior and posterior cingulate, occipital and mesial temporal cortices (Figure 2, p_*FDR*_ < 0.05, adjusted for age and years of education). Regions of decreased retention were overall symmetrical.

However, in contrast, no areas of differing ^18^F-florbetapir retention were found between the Aβ−/ND− MDD patients and controls. Besides, reverse contrast revealed no areas of increased ^18^F-florbetapir retention in the SNAP MDD patients as compared with the Aβ−/ND− MDD and control groups.

### Correlation of ^18^F-Florbetapir Retention and Cognition, and Characteristics of Depression

Given Aβ differences among the groups, separate partial correlation analyses were conducted in the SNAP MDD, Aβ−/ND− MDD, all MDD, and the whole MDD and control subject groups. After controlling for age, level of education, and HVa, there were no correlations between the global cortical ^18^F-florbetapir retention and cognitive functions, including the Mini Mental State Examination (MMSE) results and each domain-specific cognitive score in each group subset. However, significant positive correlations between HVa and cognitive functions were observed in different sample groups after controlling for age, level of education, and global cortical ^18^F-florbetapir retention. Details of partial correlation coefficients are shown in Table 3.

**TABLE 3:** Correlations of cognitive functions with global cortical Aβ depositions and HVa in the different sample groups.

	SNAP MDD (*n* = 23)		Aβ−/ND− MDD (*n* = 23)		MDD (*n* = 46)		MDD and controls (*n* = 68)	
	Global Aβ	HVa	Global Aβ	HVa	Global Aβ	HVa	Global Aβ	HVa
MMSE	–0.215	0.468*	0.057	0.234	–0.013	0.388*	0.050	0.267*
Processing Speed	–0.247	0.406	–0.064	0.274	–0.087	0.388*	–0.028	0.418**
Executive	–0.434	0.072	0.164	0.395	–0.031	0.267	–0.064	0.324*
Language	–0.203	0.285	–0.174	0.603**	–0.103	0.434**	–0.253	0.372**
Visuospatial	0.126	–0.104	0.250	0.534*	0.231	0.141	0.150	0.184
Memory	–0.073	0.389	0.269	0.486*	0.085	0.412**	0.070	0.416**
Attention	0.036	0.310	–0.325	0.392	–0.044	0.351*	–0.127	0.381**

SNAP, suspected non-Alzheimer’s disease pathophysiology; MDD, major depressive disorder; ND, neurodegeneration; HVa, adjusted hippocampal volume; MMSE, Mini Mental Status Examination. *p < 0.05, **p < 0.01 after adjustment for age, years of education and global cortical Aβ burden.

The global ^18^F-florbetapir retention was not significantly correlated with HAM-D (*r* = −0.149, *p* = 0.336), age at onset of depression (*r* = −0.084, *p* = 0.587), number of major depressive episodes (*r* = 0.037, *p* = 0.812), or time since onset of depression (*r* = 0.064, *p* = 0.682), after controlling for age and level of education.

## Discussion

Unexpectedly, the SANP MDD group not only met the predefined criteria of an amyloid-negative status, but even exhibited a significantly reduced cerebral Aβ burden relative to the control and Aβ−/ND− MDD subjects in several brain regions. The most prominent decrease emerged in the bilateral mesial temporal cortex. The lack of differences of Aβ burden in the parietal and precuneus cortex between the SNAP MDD and control groups supported that the SNAP MDD patients had no preexisting early AD pathophysiology into the amyloid pathway. HVa atrophy, but not Aβ burden, had significant correlations with cognitive deficits in the total MDD and Aβ−/ND− MDD samples. However, in the SNAP MDD patients, neither HVa nor Aβ deposition were correlated with cognitive functions.

In the context of depression increasing the risk of incident dementia, early evidence connecting Aβ to depression came from postmortem studies of AD dementia patients, revealing an association between Aβ plaque and a lifetime history of MDD (Rapp et al., 2006). The advances of validated amyloid PET imaging enabled study of Aβ *in vivo*. In our previous studies (Wu et al., 2014, 2016), elderly patients with lifetime MDD, especially those with amnestic mild cognitive impairment, carried a greater Aβ burden in the parietal and precuneus cortex as compared with the controls. However, among subjects with midlife MDD, Madsen et al. (2012) demonstrated no global Aβ difference between cognitively normal MDD patients and control subjects. A population-based longitudinal study performed in Rotterdam (Mirza et al., 2016) provided some insight into the inconsistent results regarding Aβ in depression in old age, as that study uncovered that a substantially higher risk of dementia appeared in elderly depressed groups with an increasing depression trajectory, suggesting that depression might be a prodrome of dementia. In response to the postulation regarding depression as an AD prodrome, longitudinal studies have identified incident depressive symptoms corresponding to the baseline Aβ burden in cognitively normal older adults (Harrington et al., 2017; Donovan et al., 2018; Gatchel et al., 2019). Taken together, current evidence indicates that depression, especially late-onset depression, appears more likely to be a marker of incipient dementia than a true risk factor.

Challenging existing assumptions, Mackin et al. (2021) presented a shocking finding contrary to expectations, in that depressed elderly patients showed less Aβ accumulation than non-depressed subjects. Compared with the non-depressed group, which included individuals with a matched proportion of MCI, the depressed group exhibited decreased global Aβ accumulation and a lower proportion of Aβ positivity. They performed a second similar analysis restricting the sample to subsets of cognitively normal participants both in the depressed and non-depressed groups, which yielded results indicating a significant difference with regards to Aβ positivity, but not for Aβ burden.

In the present study, we observed that the amyloid-negative MDD patients had a significantly lesser Aβ burden than the control subjects. Moreover, the SNAP MDD population was the group that contributed most strongly to the result of reduced Aβ deposition. Corresponding to the reports by Mackin et al. (2021), our findings might provide a possibility as well as evidence to explain that other non-amyloid-mediated pathways may be associated with potential cortical Aβ reduction in depressed older adults. Nevertheless, we did not agree with the interpretation speculated by Mackin et al. that reduced cerebral blood flow or hypometabolism in depression may limit regional Aβ uptake (Mackin et al., 2021). Regional cerebral hypometabolism in SNAP by definition would mimic the metabolism pattern in AD; however, AD patients present with a typical abundant Aβ burden, and therefore decreased Aβ accumulation in the SNAP MDD group might be related to their own pathology-specific factors. To date, no study has investigated the characteristic Aβ patterns showing reduced Aβ depositions in SNAP patients with or without depression (Jack et al., 2012; Burnham et al., 2016).

Even though our findings demonstrated that other non-amyloid-mediated pathways were likely associated with reduced Aβ in the SNAP MDD patients, we did not refute the possibility that an increased Aβ burden is associated with the AD pathway in depression in old age. We very much agree with the comments of Christopher et al. (Van Dyck et al., 2021) that these diverse results should remind us of the tremendous heterogeneity regarding neurodegenerative pathophysiology in depression in old age. We should not expect most of depression in old age to have a uniform relationship with dementia pathogenesis. Some depressed individuals who enter the Aβ cascade in preclinical or prodromal AD stages may carry a greater Aβ burden; other depressed individuals have diverse Aβ depositions when they are on other non-AD pathways. Global cortical Aβ deposition was not found to be associated with any neuropsychological tests, whether in SNAP, Aβ−/ND− MDD, or all amyloid-negative MDD patients. However, HVa was positively associated with several neuropsychological tests in the amyloid-negative MDD sample, and when restricting the MDD sample to the Aβ−/ND− MDD patients. These correlations disappeared when further restricting the sample to the SNAP MDD subjects, the lack of a correlation in the SNAP MDD sample perhaps being associated with unrevealed factors other than Aβ and HVa.

Several limitations of this study must be acknowledged. First, this study was limited to small sample sizes; however, our results remained robust, even when using atrophy-corrected PVC data and a stringent threshold of FDR for multiple comparisons. Second, there are amyloid-positive individuals in the cognitively healthy general population, but we included control subjects with the Aβ−/ND− profile for group comparisons on the same amyloid-negative basis. However, the SNAP MDD patients exhibited much lower Aβ depositions, while amyloid-positive control subjects were also included in the study. Third, although atrophy and hypometabolism are widely recognized as ND biomarkers in relevant SNAP studies (Caroli et al., 2015; Jack et al., 2016; Mormino et al., 2016), an operational standardized approach to measurement remains lacking a consensus (Jack et al., 2012). Individuals may be misclassified due to arbitrary cut-off values. Moreover, the pathophysiology of SNAP should remain heterogeneous. Other non-amyloid neuropathological changes, such as Aβ-independent tauopathy, hippocampal sclerosis with TDP-43, α-synucleinopathy, or argyrophilic grain disease, might contribute to and coexist with each other (Wirth et al., 2013; Mormino et al., 2016; Villeneuve, 2016). Data regarding specific pathologies of the SNAP subjects were unavailable in this study. Finally, interpretation and generalization of the findings to non-depressive subjects must acknowledge the potential limitations of this study. Notably, the finding that the SNAP and Aβ-/ND-MDD groups differed in amyloid burden despite similar psychopathological features between these two depressed groups, supported that SNAP, but not depression, is associated with reduced amyloid burden. Future studies in non-depressed subjects with SNAP are critical to determine the role of isolated SNAP on brain Aβ changes.

## Conclusion

In this study, the amyloid-negative MDD patients had a significantly lesser Aβ burden than the control subjects. The SNAP MDD patients were the group that contributed most strongly to the result of reduced Aβ deposition. SNAP MDD subjects represent a distinct study population with ND biomarkers mimicking AD, but pathological biomarkers not doing so. Our findings might provide a possible evidence that other non-amyloid-mediated pathways may be involved in underlying cortical Aβ reduction in depressed older adults. We envisage that the currently labeled entity “amyloid-negative individuals” might be further refined to discern individuals with substantially low amounts of Aβ. Meaningfully low amounts of subclinical Aβ might provide critical information on the pathogenesis of non-AD individuals.

## Data Availability Statement

The data that support the findings of this study are available from the corresponding author upon reasonable request.

## Ethics Statement

The studies involving human participants were reviewed and approved by Chang Gung Medical Foundation Institutional Review Board. The patients/participants provided their written informed consent to participate in this study.

## Author Contributions

K-YW, K-JL, and I-TH designed the study. K-YW, K-JL, I-TH, C-HC, Y-MW, and C-YL acquired the data. K-YW, K-JL, Y-MW, T-CY, C-SC, and I-TH analyzed the data. K-YW, K-JL, T-CY, and I-TH wrote the manuscript. All authors revised and approved the article for publication.

## Conflict of Interest

The authors declare that the research was conducted in the absence of any commercial or financial relationships that could be construed as a potential conflict of interest.

## Publisher’s Note

All claims expressed in this article are solely those of the authors and do not necessarily represent those of their affiliated organizations, or those of the publisher, the editors and the reviewers. Any product that may be evaluated in this article, or claim that may be made by its manufacturer, is not guaranteed or endorsed by the publisher.

## Footnotes

1
https://surfer.nmr.mgh.harvard.edu/

2
https://www.fil.ion.ucl.ac.uk/spm/
