# Supplementary material for: Decreased Cerebral Amyloid-β Depositions in Patients With a Lifetime History of Major Depression With Suspected Non-Alzheimer Pathophysiology
Source: Front Aging Neurosci. 2022 May 26;14:857940. doi: 10.3389/fnagi.2022.857940 (PMC9204309; doi:10.3389/fnagi.2022.857940)
Supplement: Supplementary file 1 [file Data_Sheet_1.pdf]

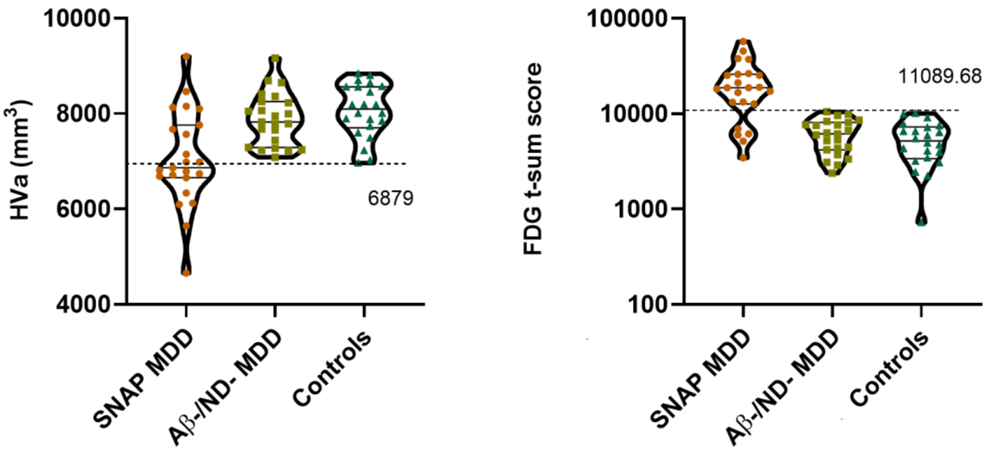

Supplementary Figure 1. Neurodegeneration biomarker distributions in the SNAP MDD (*n*=23), Aβ-/ND- MDD (*n*=23) patients, and the Aβ-/ND- control subjects (*n*=22).

Supplementary Table 1. ROI group comparisons between the amyloid-negative MDD patients and control subjects.

Characteristic	Aβ- MDD<br>*n* = 46	Controls<br>*n* = 22	p value
Frontal	1.11 ± 0.09	1.20 ±0.09	<0.001
Anterior cingulate	1.21 ± 0.15	1.36 ±0.12	<0.001
Posterior cingulate	1.29 ± 0.13	1.39 ±0.16	0.012
Occipital	1.23 ± 0.09	1.29 ±0.10	0.011
Parietal	1.12 ± 0.12	1.15 ±0.08	0.439
Precuneus	1.09 ± 0.10	1.11 ±0.08	0.586
Temporal	1.05 ± 0.09	1.14 ±0.10	0.003
Global cortex	1.11 ± 0.08	1.19 ±0.08	0.001

Mesial temporal	1.00 ± 0.09	1.08 ±0.06	<0.001
Hippocampus	1.05 ± 0.12	1.15 ±0.09	0.001
Basal ganglia	1.10 ± 0.12	1.22 ±0.09	<0.001

Abbreviations: ROI: region of interest; MDD: major depressive disorder.

p values denote the significance of differences between the MDD patients and the control subjects using the Mann-Whitney U test.

Supplementary Table 2. ROI group comparisons among the SNAP MDD, Aβ-/ND- MDD, and control groups using non-PVC original data.

Characteristic	SNAP (Aβ-/ND+) MDD<br>*n* = 23	Aβ-/ND- MDD<br>*n* = 23	Controls<br>*n* = 22	p value	Post-hoc analyses		
					SNAP vs. Controls	A-N-MDD vs. Controls	SNAP vs. A-N-MDD
Frontal	1.03 ±0.10	1.09 ±0.06	1.14 ±0.08	<0.001	<0.001	0.156	0.091
Anterior cingulate	1.12 ±0.15	1.22 ±0.08	1.29 ±0.09	<0.001	<0.001	0.094	0.068
Posterior cingulate	1.22 ±0.11	1.32 ±0.11	1.35 ±0.14	0.003	0.003	>0.999	0.032
Occipital	1.16 ±0.09	1.23 ±0.07	1.24 ±0.10	0.007	0.008	>0.999	0.061
Parietal	1.03 ±0.10	1.12 ±0.10	1.08 ±0.09	0.012	0.216	0.78	0.009
Precuneus	1.03 ±0.10	1.06 ±0.06	1.05 ±0.08	0.701	-	-	-
Temporal	0.99 ±0.09	1.04 ±0.06	1.09 ±0.08	0.001	0.001	0.066	0.43
Global cortex	1.08 ±0.09	1.15 ±0.05	1.18 ±0.08	0.001	0.001	0.543	0.043
Mesial temporal	0.96 ±0.11	1.03 ±0.07	1.07 ±0.06	<0.001	<0.001	0.43	0.035
Hippocampus	1.00 ±0.12	1.09 ±0.09	1.14 ±0.08	<0.001	<0.001	0.361	0.047

Basal ganglia	1.03 ±0.14	1.12 ±0.08	1.16 ±0.09	0.002	0.001	0.273	0.172

Abbreviations: ROI: region of interest; SNAP: suspected non-Alzheimer disease pathophysiology; MDD: major depressive disorder; ND: neurodegeneration; PVC: partial volume correction.

p values denote the significance of differences among the SNAP MDD, Aβ-/ND- MDD and control groups using the Kruskal-Wallis test.

Supplementary Table 3. ROI group comparisons among the SNAP MDD, Aβ-/ND- MDD, and control groups using ANCOVA.

Characteristic	SNAP (Aβ-/ND+) MDD *n* = 23	Aβ-/ND- MDD *n* = 23	Controls *n* = 22	p value	Post-hoc analyses		
					SNAP vs. NC	A-N-MDD vs. NC	SNAP vs. A-N-MDD
Frontal	1.06 ±0.10	1.15 ±0.06	1.20 ±0.09	<0.001	<0.001	0.302	0.003
Anterior cingulate	1.14 ±0.16	1.27 ±0.11	1.36 ±0.12	<0.001	<0.001	0.236	0.006
Posterior cingulate	1.23 ±0.13	1.35 ±0.11	1.39 ±0.16	0.001	0.001	>0.999	0.007
Occipital	1.19 ±0.10	1.27 ±0.07	1.29 ±0.10	0.001	0.003	>0.999	0.007
Parietal	1.08 ±0.12	1.16 ±0.11	1.15 ±0.08	0.015	0.081	>0.999	0.022
Precuneus	1.06 ±0.11	1.11 ±0.07	1.11 ±0.08	0.088	-	-	-
Temporal	1.01 ±0.09	1.09 ±0.07	1.14 ±0.10	0.001	0.001	>0.999	0.011
Global cortex	1.07 ±0.09	1.15 ±0.05	1.19 ±0.08	<0.001	<0.001	0.74	0.002
Mesial temporal	0.95 ±0.09	1.05 ±0.06	1.08 ±0.06	<0.001	<0.001	0.739	<0.001
Hippocampus	1.00 ±0.11	1.11 ±0.09	1.15 ±0.09	<0.001	<0.001	>0.999	0.001

Basal ganglia	1.05 ±0.12	1.16 ±0.08	1.22 ±0.09	<0.001	<0.001	0.336	0.002

Abbreviations: ROI: region of interest; SNAP: suspected non-Alzheimer disease pathophysiology; MDD: major depressive disorder; ND: neurodegeneration; ANCOVA: analysis of covariance.

[a] ANCOVA was conducted to compare regional ^{18}F-florbetapir SUVRs among the three groups using age and years of education as covariates; pairwise differences among the adjusted means were further evaluated with Bonferroni correction.
